# Progrip versus ProFlor: two fixation-free devices for laparoscopic inguinal hernia repair—the Pro/Pro study, a randomized clinical trial

**DOI:** 10.1007/s00464-025-11680-x

**Published:** 2025-04-01

**Authors:** Giuseppe Di Buono, Giorgio Romano, Vito Rodolico, Giuseppe Amato, Guido Zanghì, Giorgio Romano, Pietro Giorgio Calò, Antonino Agrusa

**Affiliations:** 1 Department of Precision Medicine in Medical, Surgical and Critical Care (Me.Pre.C.C.), University of Palermo, Via del Vespro, 129, 90127 Palermo, Italy; 2 Department PROMISE, Section Pathological Anatomy University of Palermo, Palermo, Italy; 3 Department of General Surgery, University of Catania, Catania, Italy; 4 Postgraduate School of General Surgery, University of Palermo, Palermo, Italy; 5 Department of Surgical Sciences, University of Cagliari, Cagliari, Italy

**Keywords:** Inguinal hernia, Laparoscopy, Mesh, Laparoscopic hernia repair, Hernia surgery, Dynamic scaffold

## Abstract

**Trial design:**

This randomized, multicenter clinical trial evaluates laparoscopic bilateral inguinal hernia repair outcomes by comparing the Progrip self-fixating mesh to the ProFlor 3D dynamic regenerative scaffold.

**Methods:**

Adults aged 18–85 years with clinically diagnosed bilateral primary inguinal hernias were enrolled and randomized into two treatment groups (Progrip or ProFlor) using block randomization. Operative time, intra- and postoperative complications, recurrence rates, postoperative pain, and quality of life were assessed over a 24-month follow-up. The allocation was not blinded to investigators or patients.

**Results:**

From January 2021 to June 2022, 150 patients underwent laparoscopic TAPP repair. Eighteen were lost to follow-up (5 in the ProFlor group, 13 in the Progrip group). A total of 132 patients (67 ProFlor, 65 Progrip) were analyzed. The ProFlor group demonstrated shorter operative times, fewer intra- and postoperative complications, and reduced early postoperative pain compared to the Progrip group. Patients in the ProFlor arm achieved faster recovery and earlier return to daily activities. Notably, none of the ProFlor patients experienced chronic pain, whereas 10.8% of Progrip patients developed this complication. Hernia recurrence was observed in 2 Progrip patients, while no recurrences were reported in the ProFlor™ group.

**Conclusion:**

In this randomized trial, both devices proved feasible and effective for laparoscopic repair of bilateral inguinal hernias. However, the ProFlor scaffold was associated with reduced postoperative pain, absence of chronic pain, and no recurrences during follow-up compared to Progrip. While these findings are encouraging, further studies with larger cohorts and longer-term follow-up are warranted to confirm the potential benefits of the ProFlor scaffold and its role in routine clinical practice.

Trial registration: This study was registered at ClinicalTrials.gov with number NCT06556498.

**Supplementary Information:**

The online version contains supplementary material available at 10.1007/s00464-025-11680-x.

Plenty of techniques and a variety of implants are employed in the treatment of inguinal hernias. The practice of reinforcing the herniated groin with a flat mesh has proven effective in mitigating the recurrence rate prevalent in earlier pre-prosthetic methods [1]. Besides open groin hernia repair, laparoscopic techniques are widely utilized. Nevertheless, a definitive gold standard for hernia treatment remains elusive [2, 3].

Over the past two decades, laparoscopic repair has gained prominence, particularly for recurrent cases or bilateral hernias following open anterior repair [4]. However, laparoscopic TAPP and TEP surgeries pose challenges related to anatomy, technique, and prosthetic selection [5].

For large defects in laparoscopic groin hernia repair, larger and heavier meshes with multiple fixation devices are employed to prevent mesh invagination under abdominal pressure. This necessitates broader peritoneal dissection and heightens the risk of complications [5, 6]. Such an approach often leads to the formation of fibrotic bridles and scar tissue, contributing to postoperative pain and potential chronic pain syndrome [7–15].

In TEP and TAPP laparoscopic procedures, commonly metallic or resorbable tacks are employed for mesh fastening. However, this practice can adversely affect abdominal wall physiology. The insertion of screwed tacks, which sharply penetrate the delicate muscular surroundings, inherently disrupts groin kinetics, potentially leading to complications [16, 17]. Furthermore, in cases where the hernia opening exceeds 2 cm, visceral pressure may displace the implant toward this vulnerable area, increasing the risk of mesh mobilization and invagination into the defect. These issues represent a primary cause of recurrence in laparoscopic inguinal hernia repair [6].

To address these shortcomings, extensive research has been conducted to innovate and produce new devices aimed at achieving fixation-free repairs. One notable advancement arises from the development of a composite mesh incorporating monofilament polyester fabric and a resorbable polylactic acid gripping system. This bicomponent mesh, known as Progrip, is designed for secure, fixation-free placement in the posterior aspect of the inguinal wall. It offers a promising solution to circumvent complications associated with the use of fixation tools on inguinal tissues and nerves, which can contribute to discomfort and the development of chronic pain syndrome [18–20].

However, despite its promising aspects, the Progrip concept does not address the management of hernia defects that persist as patent after mesh deployment. Specifically, while Progrip relies on the biological response of scar tissue formation to strengthen the groin, it leaves the hernia defect itself open. This approach does not directly seal or obliterate the opening, creating a risk of recurrence. Over time, abdominal pressure and movement may cause the mesh to shift or invaginate into the patent defect, allowing protrusion of abdominal contents. This limitation underscores a critical need for a repair strategy that not only reinforces the surrounding tissue but also provides a complete and permanent obliteration of the hernia defect. Such an approach is vital to addressing one of the primary causes of recurrence following laparoscopic hernia repair [21].

To tackle these challenges head-on, a novel treatment approach has surfaced: the utilization of a 3D dynamic responsive scaffold named ProFlor. This approach is designed to achieve permanent closure of the hernia opening, providing a potential strategy for hernia repair [22–24]. According to the manufacturer, this 3D hernia device, made from polypropylene, is designed to be spring-like and requires no fixation, purportedly moving with the groin and promoting a probiotic biological response [25, 26].

ProFlor is employed for both open and laparoscopic hernia repair, with the aim of further minimizing surgical trauma and ensuring permanent defect obliteration [27, 28]. Scientific studies have demonstrated the development of connective tissue, vessels, nerves, and muscles within the 3D scaffold, attributing these regenerative effects to its dynamic responsivity [29–34].

In summary, two methods currently exist for fixation-free laparoscopic inguinal hernia repair, specifically based on the intrinsic properties of the device used. The Progrip mesh technique leaves the hernia orifice patent and relies on the established principle of strengthening the groin through scar tissue incorporation induced by foreign body reaction. In contrast, the ProFlor concept introduces a 3D dynamic regenerative scaffold that permanently obliterates the defect and regenerates the herniated inguinal barrier.

This randomized clinical trial evaluates adult patients with bilateral primary inguinal hernias undergoing laparoscopic repair, comparing the Progrip self-fixating mesh to the ProFlor 3D dynamic regenerative scaffold, with outcomes measured in terms of operative time, complications, recurrence rates, postoperative pain, and quality of life over 24 months. This randomized clinical trial is reported in line with CONSORT guidelines [35].

## Material and methods

Institutional ethics committee approved the study (protocol number 17/2024) that was designed as a multicentric randomized controlled trial (Pro/Pro Study) with parallel arms and allocation ratio of 1:1. The Ethical approval contains the entire protocol of this study that was also registered at ClinicalTrials.gov with number NCT06556498.

### Patients’ selection (Fig. 1)

**Fig. 1:**
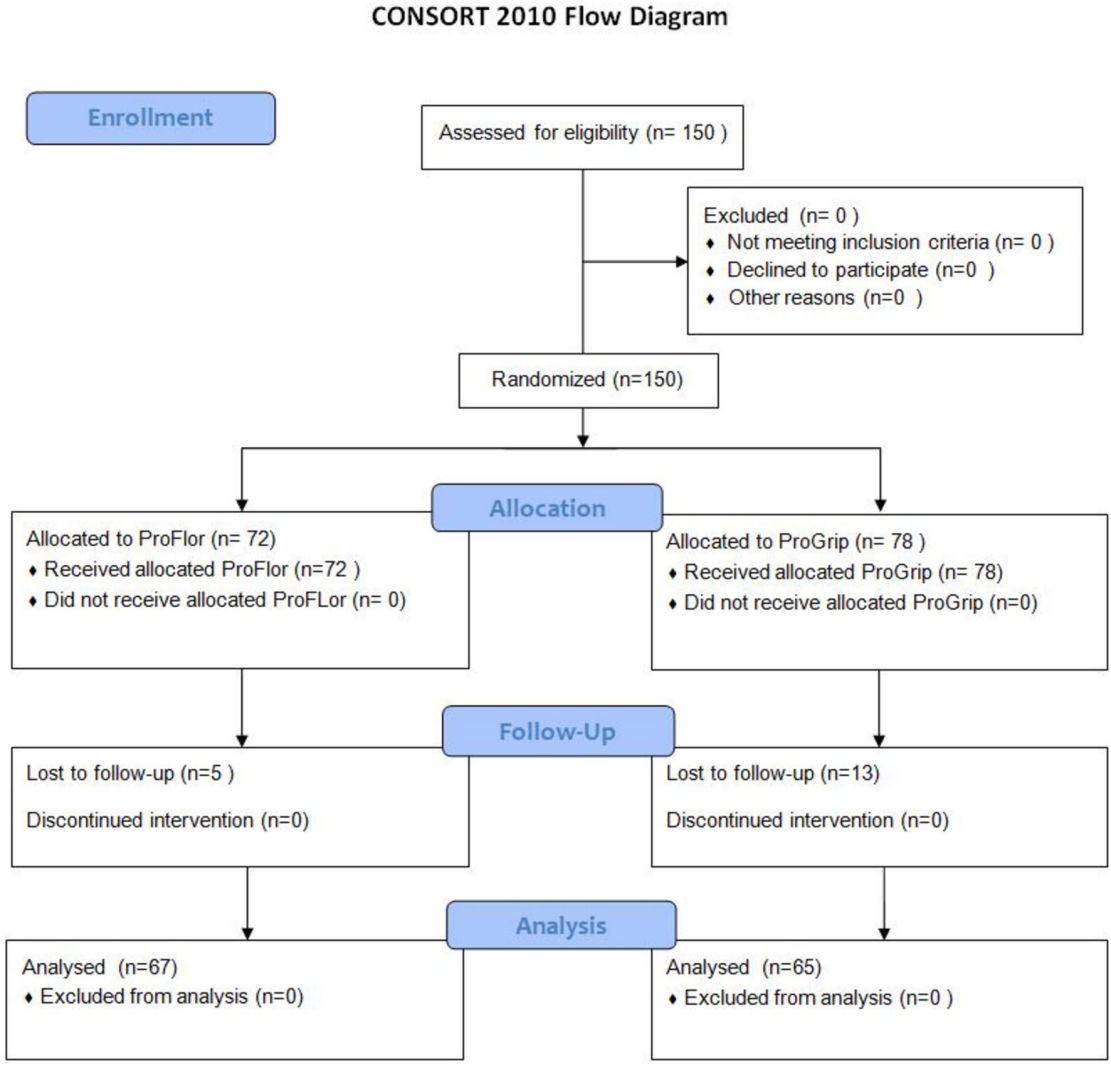
CONSORT 2010 flow diagram

Patients competent to give consent, aged between 18 and 85 years, were included in the report. All individuals were diagnosed with clinically relevant bilateral inguinal hernia. We excluded recurrent inguinal hernia, because we can test the different response to prosthesis in primary hernia. Exclusions criteria are enlisted in Table 1. The patients were randomly assigned to the two parallel arms of treatment in a 1:1 ratio with a block randomization performed by our department at the University of Palermo with the computer-based random number method. After randomization, the information regarding planned procedures was sent to the surgical team the day before surgery. The allocated procedure was not concealed to the investigators or patients. All patients of both groups signed a specific informed consent. Preoperative assessment of the hernia opening carried out with US and/or CT scans was considered mandatory for all patients. Specifically for the ProFlor procedure, the scope of the measurement was to preoperatively define the size of the scaffold core to be chosen.

**Table 1:** Exclusion criteria

Exclusion criteria
Recurrent inguinal hernia
Incarcerated inguinal hernia
Hernia not in the inguinal area
Signs of obvious local or systemic infection
ASA score > 4
Presenting with unstable angina or NYHA class of IV
Pregnant
Active drug user
Immunosuppression, chemotherapy
Presenting with unstable angina or NYHA class of IV
Chronic renal insufficiency
Abdominal ascites
Infection in area of the surgical field
BMI > 34

### Hernia devices

**Fig. 2:**
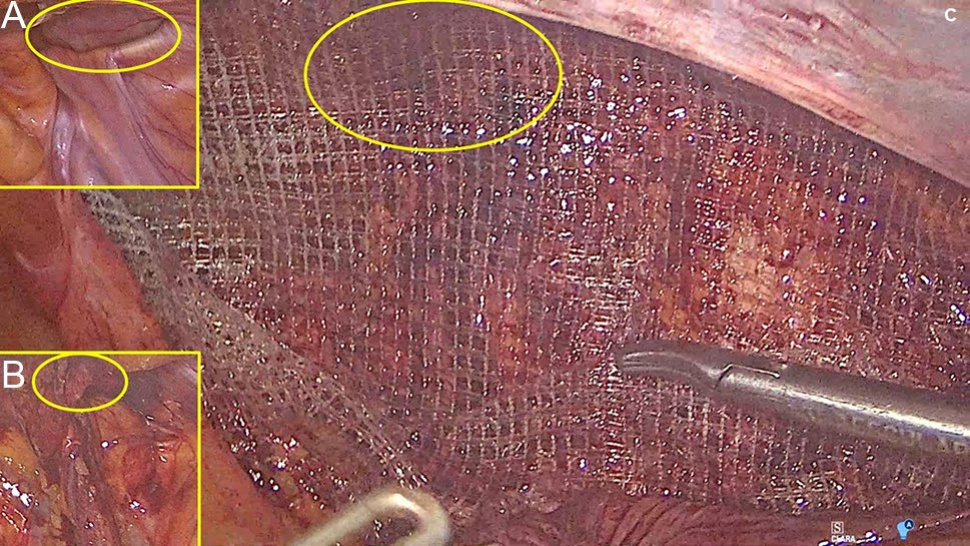
**A** Intra-abdominal view of indirect inguinal hernia defect right (yellow circle). **B** Preperitoneal view of the hernia defect (yellow circle) after peritoneal dissection. **C** Progrip laparoscopic self-fixating mesh deployed over the inguinal backwall and covering the hernia defect (yellow circle). Of note, the great amount of thick prosthetic material covers the posterior inguinal area (Color figure online)

**Fig. 3:**
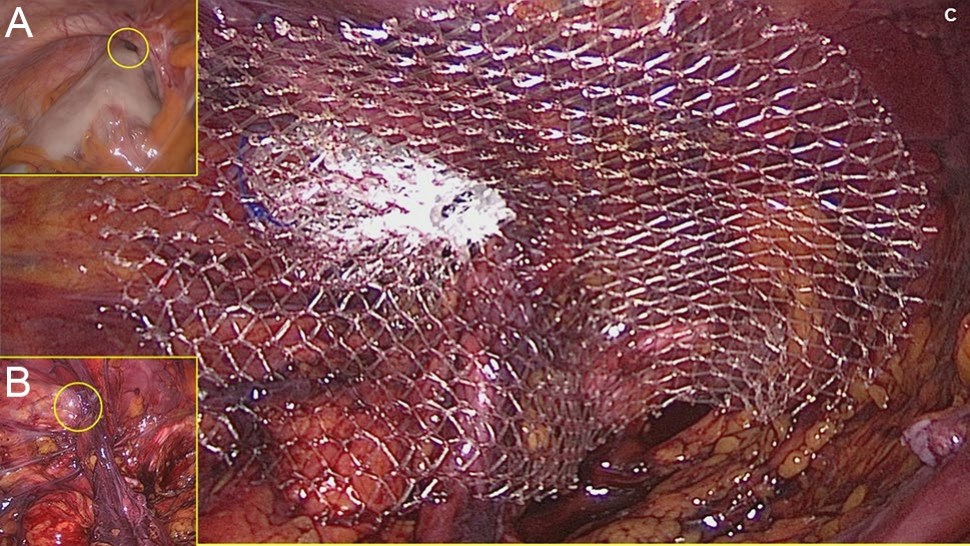
**A** Intra-abdominal view of indirect inguinal hernia defect left (yellow circle) with involvement of the sigma. **B** Preperitoneal view of the hernia defect (yellow circle) after peritoneal dissection. **C** The squeezed 3D scaffold of a 40-mm ProFlor E fully obliterates a the indirect hernia opening while its flat part with large pores prophylactically covers the medial, the supravesical, and the femoral fossa (Color figure online)

Two devices specifically produced for fixation-free inguinal hernia repair were used for the investigation: Progrip and ProFlor. Progrip, laparoscopic self-fixating mesh produced by Medtronic (USA) is a flat and static mesh made of macroporous, monofilament polyester material and provided on one surface by thousands microhooks of absorbable polylactic acid (Fig. 2). Said flat mesh is positioned over the herniated groin and covers the defect. The integrated hooks confer to the implant self-gripping properties and are over time absorbed. Since the microhooks stick over the inguinal tissue, no fixation is needed to hold the mesh in place. In contrast, ProFlor E (with extended flat part used for this study), produced by Insightra Medical Inc., USA, exhibits different designs. It is a dynamic responsive scaffold made of knitted lightweight, light porous polypropylene monofilament having pores sized 2.8 × 2.6 mm and weighting 88 g/m^3^. Featuring a multilamellar cylindrical 3D structure with reinforced edges, ProFlor E comes in two sizes: 25 and 40 mm in diameter and each with a thickness of 15 mm. At the center of the core of the scaffold lies on one surface an oval-shaped flat mesh measuring 8 × 10 cm, designed to cover the inguinal and femoral area while opposing the peritoneal sheath (Fig. 3). The 3D core of the scaffold is flexible in both longitudinal and transversal planes, allowing it to compress and expand in tandem with the movements of the groin. This inherent dynamic responsivity enables ProFlor E to adapt to the body’s motions. Moreover, its proprietary centrifugal expansion mechanism facilitates fixation-free positioning within the hernia defect, ensuring permanent obliteration.

### Surgical techniques

Perioperatively, all patients received a single dose of 2 g of Ceftriaxone intravenous as antibiotic prophylaxis. All patients underwent the TAPP surgeries under general anesthesia. Surgeon experts in laparoscopic hernia repair carried out the procedures (at least previous 50 TAPP procedure with different hernia devices). After establishing pneumoperitoneum, three trocars were positioned at the umbilical level: a 12-mm large optic trocar at the navel and two working 5-mm ports placed over the right and left rectus muscles. All trocars were carefully inserted under direct vision. Following identification of the hernial defect and retraction of visceral protrusion into the peritoneal cavity, a meticulous dissection of the parietal peritoneum was conducted to expose the inguinal backwall. Subsequently, adhesiolysis of the hernia sac was performed, and the dissected sac was returned to the abdominal cavity. Up to this point, the procedures were uniform across all patients. Upon completion of the dissection of the posterior abdominal wall, a 10 × 15 Progrip Laparoscopic Self-fixating Mesh Bilateral Anatomical was introduced through a 12-mm trocar in 78 patients. The mesh was then delivered and meticulously positioned over the inguinal backwall and covering the hernia defect. Thanks to the polylactic acid hook of the self-gripping mesh, the implant could be flattened over the inguinal tissue without need of fixation. In the remaining 72 patients, the ProFlor scaffold type E, rolled upon the flat part and compressed along the longitudinal axis, was inserted through the 12-mm trocar. Upon deployment into the abdominal cavity, the natural springiness of the 3D scaffold quickly regained its original form. Subsequently, the core of ProFlor E was introduced and meticulously deployed within the hernial defect, ensuring that its edges were aligned with the hernia borders to comprehensively seal the opening. Once the hernia device was securely placed, thanks to the centrifugal expansion of the multilamellar 3D core the scaffold expanded within the defect. At this stage, a simple test involving repeated attempts to pull out the flat part of the scaffold confirmed the permanence of the 3D core within the defect. No fixation was required to secure ProFlor E in place for any of the patients. Then, the flat part of the device was deployed over the posterior aspect to entirely cover the inguinal area. Closure of the peritoneal flap marked the completion of the procedure for all patients in both groups.

### Postoperative evaluation and outcomes

All patients enrolled in the investigation underwent comprehensive clinical evaluation scheduled at defined intervals: 7 days, 15 days, 1 month, 6 months, 12 months, and 24 months postoperatively. The primary objective of these evaluations was to detect any possible complications that may have arisen, including but not limited to recurrence, hematoma, seroma, testicular swelling, wound infection/abscesses, prosthesis displacement, or any other complication. During follow-up control, patients were tasked with completing questionnaires aimed at evaluating their clinical conditions. To assess pain levels during the early stage (until four-week postoperative), the Visual Analogue Score (VAS) was employed. The VAS score is a widely recognized tool that enables patients to rate their pain on a scale from 0 to 10, where 0 signifies no pain and 10 indicates the most severe pain imaginable. Though, for long-term clinical evaluation starting from four-week postoperative, patients were instructed to fill out the Carolinas Comfort Scale questionnaire (CCS), regarded as the optimal scoring system for assessing pain, discomfort, and other postoperative symptoms. The CCS questionnaire offered a more thorough insight into the patient’s experience and facilitated the evaluation of their long-term outcomes [36, 37].

### Statistical analysis

For the statistical assessment, we considered the overall incidence of postoperative adverse events as primary endpoint across the two arms. The collected data were divided into two phases: the early and the late postoperative phase. The secondary endpoints included evaluations of postoperative pain, discomfort, and quality of life. These secondary endpoints were assessed over two distinct periods. The initial period encompassed the early postoperative phase, lasting up to one month postoperatively, during which pain levels were measured using the Visual Analogue Scale (VAS) scoring system. The second period covered the late postoperative stage, extending from one-month to two-year post-surgery. During this time, postoperative pain and the patient’s quality of life were assessed using the Carolinas Comfort Scale (CCS).

The statistical analysis was performed using SPSS 25.0 (SPSS Inc., Chicago, IL, USA). We used Shapiro–Wilk test to define the normal distribution of the data. Quantitative variables with normal distribution were expressed as mean and standard deviation (SD) and compared using the Student’s *t* test. Categorical variables were presented as counts and percentages and compared using the Chi-squared test. A *P* value less than 0.05 was considered statistically significant. Prior to patient enrollment in the two groups, a thorough power analysis was conducted to ensure the study possessed adequate statistical power and to mitigate the risk of Type II errors. The sample size was calculated based on the review of existing literature concerning laparoscopic inguinal hernia repair and our preliminary data regarding the rate of overall postoperative complications. This percentage ranges from 1% in the case of recurrences up to 35% in the case of seromas [38–40]. On the basis of these results, the power analysis revealed that a minimum of 74 cases of hernia repair for arm would be needed to detect significant differences of the clinical outcomes. Specifically, this sample size was calculated to identify a 5% comprehensive reduction of the adverse events (bleeding, hematoma, and infections) between the two groups and a decrease of 2 points in the VAS score for postoperative results. The 132 patients of this study underwent bilateral inguinal hernia repair, resulting in a total of 234 hernia repair cases evaluated in the statistical analysis. With a significance level set at 5% and a power calculation of 80%, the investigation aimed to confirm that the sample size was adequate to detect substantial differences between the two arms of the study, thereby yielding consistent and significant results.

## Results

Between January 2021 and June 2022, a total of 150 patients were included in the investigation and operated for inguinal hernia with the TAPP technique. 78 patients formed the subset of individuals operated with the Progrip mesh and the remaining 72 underwent hernia repair with the laparoscopic ProFlor approach. During follow-up, 18 patients were lost, 5 from the ProFlor group and 13 patients in the Progrip group. For the statistical analysis were enrolled in total 132 patients: 65 in the Progrip arm and 67 patients in the ProFlor group (Fig. 1). Concerning demographics, all the patients were male. The mean age of the patients was 52.3 ± 24.5 years. 87 patients (66%) suffered by comorbidities, mainly cardiovascular, metabolic, or pulmonary diseases, with no significant differences between the two arms. Additional info about demographics, comorbidities and ASA scores are highlighted in Table 2. Concerning the classification of the hernias managed during the study, since available classification models do not comprise relatively frequent hernia types, such as supravesical hernias and multiple ipsilateral, the encountered hernias were categorized following a new updated classification model that also includes these often misidentified or overlooked protrusions [41]. Concerning classification, we considered 132 patients with a total of 264 cases of inguinal hernia. Specifically, the hernia types observed in the Progrip group were as follows: 64 cases with indirect inguinal hernias, 31 cases of direct hernias, 19 cases of multiple ipsilateral hernias, 11 cases of combined protrusions, and 5 cases of supravesical hernias. In the ProFlor group, 62 cases of indirect hernias, 23 cases of direct hernias, 25 cases of multiple ipsilateral hernias, 16 cases of combined hernias, and 8 cases with supravesical protrusions. Surgical time length for the Progrip arm had a mean operative time of 78 ± 38.6 min, while the ProFlor group had a mean duration of 65 ± 29.2 min (*P* value 0.516). Additional information concerning the defect size, hernia types and the time length of the procedures are highlighted in Table 3. Concerning intraoperative adverse events, during the Progrip hernia repair, only one patient experienced the occurrence of intraoperative bleeding, which was swiftly managed. Conversely, no intraoperative complications were encountered during ProFlor procedures. Overall, early postoperative complications, encompassing seroma, hematoma, and infections, occurred in 23 cases (35.4%) within the Progrip group and in 8 cases (11.9%) within the ProFlor group, denoting a statistically significant discrepancy (*P* value 0.035). Remarkably, no instances of implant dislodgement, recurrences, or other adverse events were documented during this early postoperative period. Concerning complications in the late postoperative stage (beyond one month), 7 patients (10.8%) who underwent Progrip hernia repair experienced chronic pain. Among these cases, 5 were resolved within six months with medical treatment, while the remaining 2 cases continued to report pain beyond the period of this investigation. No patients in the ProFlor group reported chronic pain or discomfort. No cases of testicular swelling were reported in both groups. Two patients of the Progrip arm noticed one-sided recurrence within 24-month post-surgery. Both recurrences in the Progrip arm involved a patients with hernia defects larger than 35 mm. Contrariwise, in the ProFlor cohort, no adverse events worth of note could be reported in the long term, above all no chronic pain and no cases of recurrence. Additional details concerning late complications posts-surgery are highlighted in Table 4. The ProFlor group exhibited notably less analgesic medications during the early postop stage in comparison to the Progrip group. Actually, individuals undergoing Progrip hernia repair necessitated routine pain management with 1000-mg paracetamol 3 times a day for up to 7 days post-surgery. Conversely, in the ProFlor cohort, patients received 1000-mg paracetamol three times daily for the initial three-day post-surgery. Additional analgesic supplementation was seldom required beyond the third day for ProFlor patients. The outcomes of the investigation reveal that patients undergoing the ProFlor technique reported considerably lower scores of early postoperative pain compared to those of the Progrip arm. At 1st postoperative day, Progrip patients recorded an average pain score of 4.2 on the VAS Scale, while ProFlor patients reported a higher average score of 1.6, indicating less pronounced pain. By the seventh postoperative day, Progrip patients still experienced some discomfort, with a mean VAS score of 3.1, whereas the nearly all ProFlor patients were entirely pain free, registering a mean VAS score of 0.7. These discrepancies in pain level continued even at 30-day post-surgery, where Progrip patients had an average VAS score of 1.6, while all ProFlor were pain free. All details concerning the pain scores in the early postoperative stage are highlighted in Table 5.

**Table 2:** Patients’ demographics, comorbidities, and ASA scores

	Progrip (*n*. 65)	ProFlor (*n*. 67)	Total (*n*. 132)	*P* value
Age (Mean ± SD)	Years 51.6 ± 23.2	Years 53 ± 25.4	Years 52.3 ± 24.5	0.998
Gender [*n*(%)]				
M	65 (100%)	67 (100%)	132 (100%)	1.000
F	0	0	0	–
Body Mass Index (Mean ± SD)	25.5 ± 7.3	27.3 ± 9.6	26.4 ± 8.4	0.865
Comorbidity [*n*(%)]	42 (65%)	45 (67.1%)	87 (66%)	0.865
Cardiovascular	31 (47.7%)	32 (47.7%)	63 (47.7%)	
Pulmonary	4 (6.1%)	4 (6%)	8 (6%)	
Diabetes	3 (4.6%)	5 (7.4%)	8 (6%)	
Others	4 (6.1%)	4 (6%)	8 (6%)	
ASA score [*n*(%)]				
ASA 1	23 (35.3%)	22 (32.8%)	45 (34.1%)	0.719
ASA 2	34 (52.3%)	36 (53.7%)	70 (53%)	0.998
ASA 3	8 (12.3%)	9 (13.4%)	17 (12.9%)	0.667

**Table 3:** Hernia types, defect size (total/rate), and length of the surgical procedures

	Progrip 130 inguinal defects	ProFlor 134 inguinal defects	Total 264 inguinal defects	*P* value
Hernia types [*n*(%)]				
Indirect	64 (49.2%)	62 (46.3%)	126 (47.7%)	0.719
Direct	31 (23.8%)	23 (17.2%)	54 (20.4)	0.442
Multiple ipsilateral	19 (14.6%)	25 (18.6%)	44 (16.7%)	0.676
Combined	11 (8.5%)	16 (12%)	27 (10.2%)	0.719
Supravesical	5 (3.8%)	8 (6%)	13 (5%)	0.877
Defect size (Mean ± SD)	2.6 cm ± 1.65	2.5 cm ± 1.4	2.55 cm ± 1.5	0.998
Procedure duration (Mean ± SD)	78 min ± 38.6	65 min ± 29.2	71.5 min ± 33.7	0.516

**Table 4:** Postoperative results Progrip/ProFlor compared

	Progrip (*n*. 65) *n* (%)	ProFlor (*n*. 67) *n* (%)	*P* value
Intraoperative complications	1 (1.5%) (bleeding during dissection)	–	0.998
Early complications			
Seroma	18 (27.7%)	8 (11.9%)	
Hematoma	5 (7.7%)	0 (0%)	
Wound infection	0 (0%)	0 (0%)	
Recurrence	0 (0%)	0 (0%)	
Early complications (all)	23 (35.4%)	8 (11.9)	0.035
Late complications			
Chronic pain	7 (10.8%)	0(0%)	0.001
Testicular swelling	0 (0%)	0 (0%)	
Recurrence	1 (1.5%)	0 (0%)	
Late complications (all)	8 (12.3%)	0 (0%)	0.001

**Table 5:** VAS scores at postoperative day 1, 7, and 30

VAS scores POD 1/7/30			
	Progrip (*n*. 65) Mean ± SD	ProFlor (*n*. 67) Mean ± SD	*P* value
POD 1	4.2 ± 3.1	1.6 ± 1.2	0.023
POD 7	3.1 ± 2.3	0.7 ± 0.5	0.016
POD 30	1.6 ± 1	–	0.067

The detail of the comparative total CCS scores for patients who underwent Progrip and ProFlor procedures at postoperative stages beyond one month was as follows:

- At 6-month post-surgery, the CCS score was 34.81 (mean 1.51) for Progrip patients and4.54 (mean 0.19) for ProFlor patients.
- At 12-month post-surgery, the CCS score was 11.52 (mean 0.50) for Progrip patients and 0.87 (mean 0.04) for ProFlor patients.
- At 24-month post-surgery, the CCS score was 0.77 (mean 0.03) for Progrip patients and 0.37 (mean 0.01) for ProFlor patients.

**Fig. 4:**
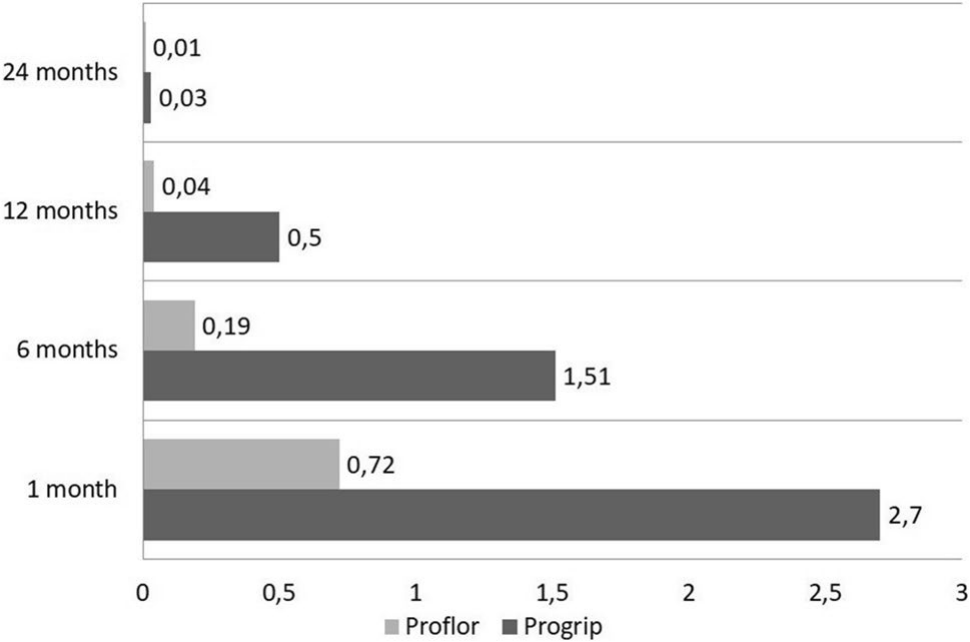
CCS score at 1, 6, 12, and 24 months compared between Progrip and ProFlor techniques

It is noteworthy that patients undergoing the Progrip technique exhibited a gradual decline in all assessed parameters, with the exception of 2 individuals who persisted with chronic pain. Nevertheless, the overall scores remained relatively elevated even after 6 months, showcasing a significant reduction only after 12 and 24 months (refer to Table 6 for detailed CCS scores). In the postoperative phase, one month following surgery, the probability of encountering symptoms in the pain and limitations subset of CCS was notably elevated in the Progrip cohort as indicated by univariate analysis. Moreover, multivariate analysis corroborated this observation, particularly for patients operated with the Progrip mesh. The average scores and the temporal trajectory of the Carolinas Comfort Scale for both arms are presented in Table 6. Notably, a CCS score below 2 is considered clinically insignificant, highlighting that the use of the ProFlor scaffold achieves this threshold within just one month postoperative. In contrast, patients undergoing the Progrip technique require six months postoperatively to achieve comparable results. This findings underscore an important clinical benefit that holds strong relevance for patient well-being, even from the early postoperative phase. Figure 4 highlights the outcomes of the CCS scoring systems.

**Table 6:** Carolinas Comfort Scale scores compared between Progrip and ProFlor at 1, 6, 12, and 24 months

	1 month Progrip/ProFlor	6 months Progrip/ProFlor	12 months Progrip/ProFlor	24 months Progrip/ProFlor
Laying down				
A. sensation of mesh	3.15/1.25	1.22/0.18	0.28/0.01	0.01/0.01
B. pain	2.67/0.52	1.08/0.20	0.13/0.01	0.02/0.01
Bending over				
A. sensation of mesh	2.56/1.0	1.31/0.18	0.55/0.02	0.05/0.00
B. pain	2.32/0.77	1.46/0.21	0.38/0.01	0.03/0.00
C. movement limitations	2.24/0.83	1.79/0.0.29	0.51/0.10	0.03/0.01
Sitting				
A. sensation of mesh	3.53/0.78	1.12/0.25	0.49/0.01	0.06/0.02
B. pain	2.16/0.65	1.23/0.24	0.42/0.08	0.04/0.03
C. movement limitations	2.42/0.72	1.65/0.22	0.56/0.06	0.07/0.02
Activities of daily living				
A. sensation of mesh	2.36/0.79	1.25/0.16	0.42/0.03	0.01/0.02
B. pain	2.87/0.65	1.74/0.24	0.38/0.03	0.04/0.01
C. movement limitations	2.98/0.74	2.04/0.25	0.32/0.12	0.06/0.02
Coughing or deep breathing				
A. sensation of mesh	2.33/0.68	1.62/0.34	0.82/0.05	0.02/0.01
B. pain	3.26/0.87	1.41/0.15	0.71/0.04	0.03/0.01
C. movement limitations	2.85/0.62	1.85/0.18	0.71/0.04	0.05/0.03
Walking				
A. sensation of mesh	3.35/0.56	1.23/0.16	0.35/0.06	0.02/0.01
B. pain	2.54/0.51	1.35/0.11	0.47/0.02	0.03/0.01
C. movement limitations	2.62/0.49	1.42/0.10	0.41/0.01	0.01/0.01
Stairs				
A. sensation of mesh	2.1/0.67	1.77/0.18	0.56/0.02	0.02/0.01
B. pain	2.75/0.59	1.43/0.15	0.52/0.01	0.01/0.01
C. movement limitations	2.63/0.51	1.32/0.15	0.58/0.01	0.03/0.02
Exercise				
A. sensation of mesh	2.13/0.51	1.82/0.19	0.89/0.01	0.07/0.06
B. pain	2.54/0.85	1.58/0.21	0.54/0.03	0.03/0.02
C. movement limitations	3.68/0.97	2.12/0.20	0.52/0.09	0.03/0.02
Overall	62.04/16.53	34.81/4.54	11.52/0.87	0.77/0.37
Mean	2.7/0.72	1.51/0.19	0.50/0.04	0.03/0.01

## Discussion

### Laparoscopic hernia repair challenges

In recent years, the laparoscopic transabdominal preperitoneal (TAPP) technique has gained significant traction for inguinal hernia repair. Like the totally extra-peritoneal (TEP) technique, the TAPP approach entails the placement of flat meshes over the herniated groin to fortify the inguinal floor. However, this concept of cure has sparked controversy due to several issues, regarding above all mesh fixation, defect patency, and the quality of biological response. Fastening the mesh in the highly sensitive and mobile muscular arrangement of the groin appears inconsistent with the dynamic environment of this area, rendering it physiologically incompatible. Extensive literature has associated implant fixation with several postoperative adverse events, including tissue tear, bleeding, hematomas, mesh dislodgement, discomfort, and chronic pain [10, 42, 43]. Of particular concern is chronic pain, which significantly affects the patient’s quality of life. [7, 11, 44].

### Fixation-free alternatives: Progrip and ProFlor

Efforts to address these issues have led researchers to explore alternatives like fibrin glue for fastening meshes. However, its high cost and short efficacy window (completely absorbed within fifteen days) have limited its utility [45–48]. This study instead focuses on two fixation-free devices, the self-fixating Progrip mesh and the 3D dynamic regenerative scaffold ProFlor, which differ significantly in material, shape, and treatment philosophy.

Progrip is a polyester-based flat mesh featuring thousands of hooks made of resorbable polylactic acid, providing self-fixating properties akin to Velcro [49]. In contrast, ProFlor is a lightweight polypropylene scaffold with a unique multilamellar 3D design that expands within the defect, eliminating the need for fixation. This study utilized the ProFlor E version with a flat mesh measuring 8 × 10 cm. This unique configuration endows the device with compressibility across all planes, enabling its insertion in a compressed state into hernia defects. Upon deployment, it leverages its inherent centrifugal force to expand and firmly anchor itself within the defect, eliminating the necessity for any kind of fixation [24].

### Comparative analysis of treatment paradigms

**Fig. 5:**
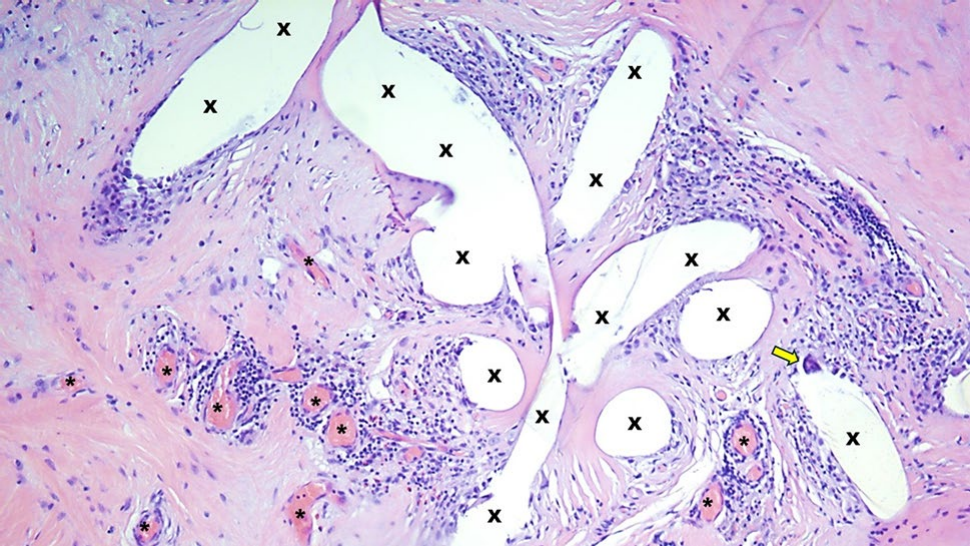
Progrip mesh explanted 10-month postop.—Implant fibers surrounded by abundant fibrous connective tissue (colored in pale red). Massive, persistent inflammatory infiltrate (dark-rounded spots) composed by lymphocytes and plasma cells close and around the implant fivers (X). A bundle of capillary vessels provide for the vascular support to the inflammation (*). A multinucleated giant cell is also detectable (yellow arrow). E&E X200 (Color figure online)

**Fig. 6:**
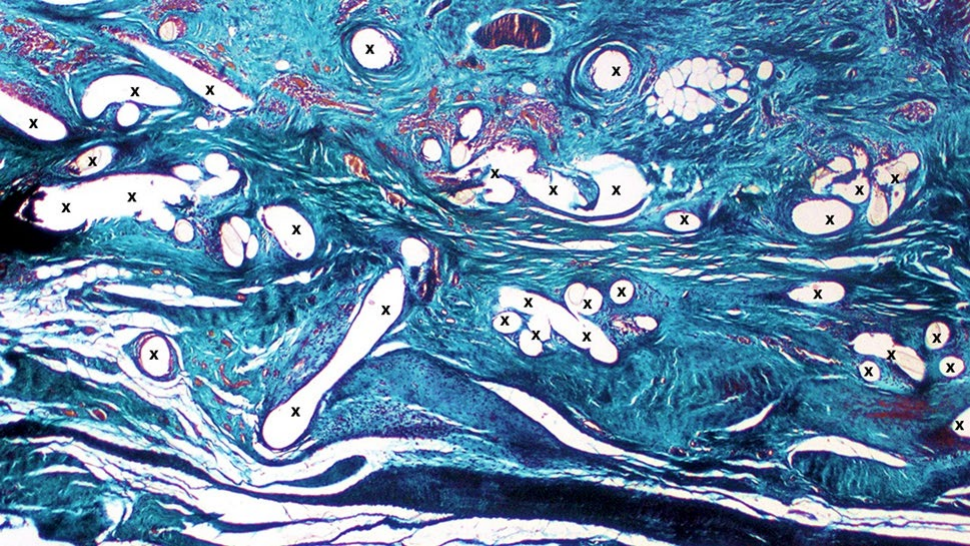
Progrip mesh explanted 8-month postop.—Implant fibers (X) surrounded by abundant fibrous connective tissue (colored in blue). Capillary vessels containing erythrocytes are also present (colored red). No muscular elements are detectable. Azan Mallory Trichrome stain X 25 (Color figure online)

**Fig. 7:**
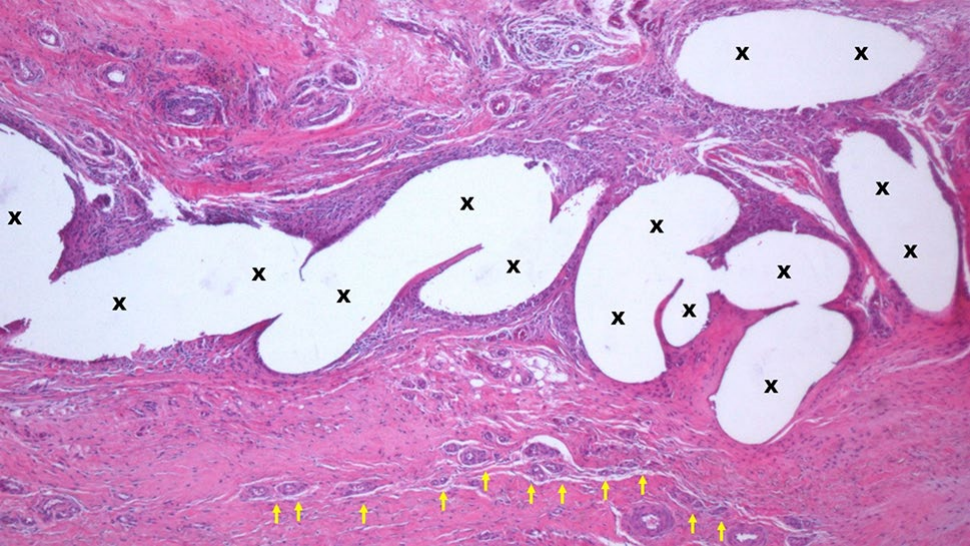
Biopsy specimen excised from ProFlor 7-month postop.—Implant fibers (X) surrounded by abundant muscular tissue (colored in red) in a contest of viable, well-hydrated connective. Numerous vascular elements are also present (white/red targeted spots). A longitudinally running nerve axon is also detectable (yellow arrows). Overall, no signs of inflammatory infiltrate. E&E X 50 (Color figure online)

**Fig. 8:**
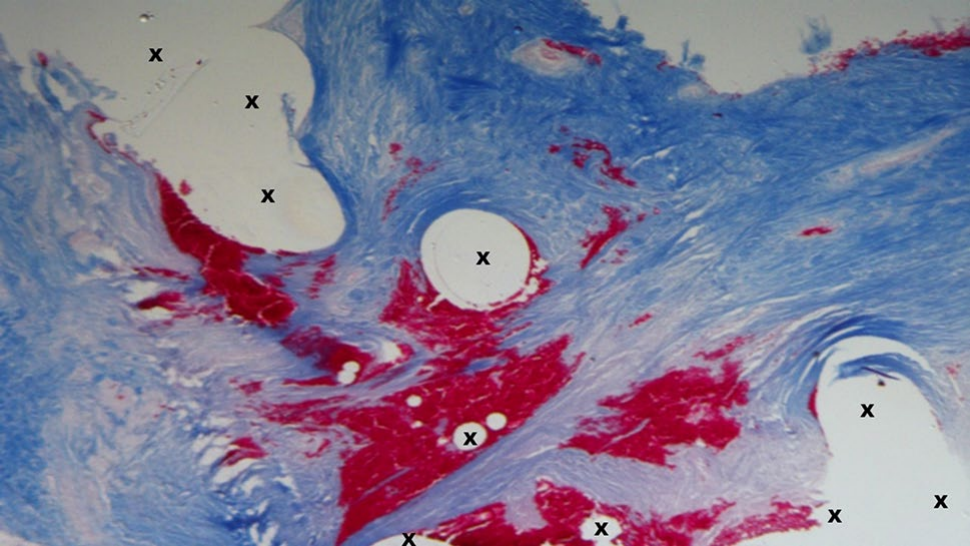
Biopsy specimen excised from ProFlor 12-month postop.—In a surround of lax and well-hydrated connective (colored in blue) are numerous bundles of muscular elements (colored in red) detectable among the fibers of ProFlor (X). Azan Mallory Trichrome × 50 (Color figure online)

**Fig. 9:**
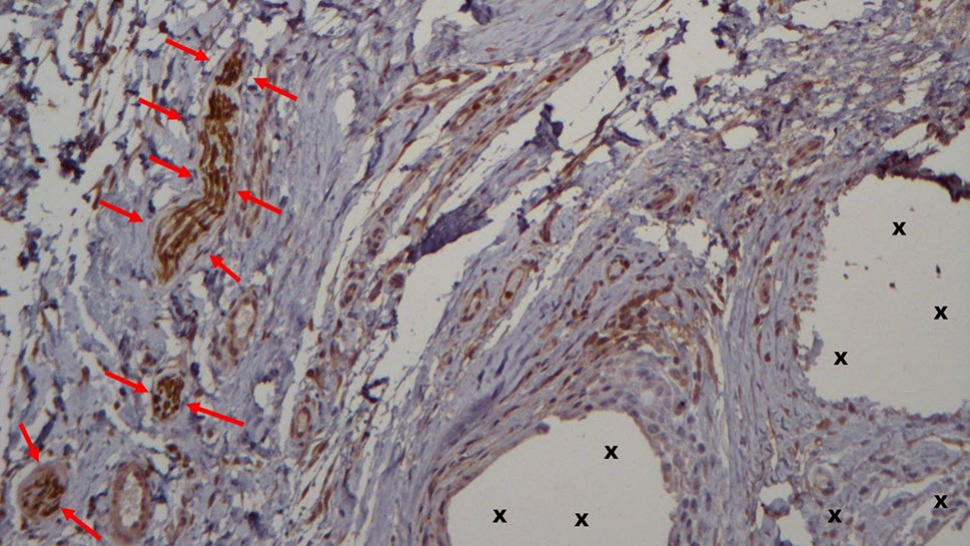
Biopsy specimen excised from ProFlor 12-months postop.—In a surround of lax and well-hydrated connective (colored in blue) are numerous bundles of muscular elements (colored in red) detectable among the fibers of ProFlor (X). Azan Mallory Trichrome × 50 (Color figure online)

Progrip and ProFlor epitomize two distinct concepts of treatment. Progrip featuring adhering hooks represents a fixation-free variation of the traditional approach aimed to reinforcing the groin with the mesh’s fibrotic incorporation as a biological response. However, akin conventional flat meshes treatment concept, it does not contemplate the management of the hernia defect, leaving it open to potential recurrent protrusion of abdominal viscera over time. From a biological standpoint, concerning the chemical composition of Progrip, which includes permanent polyester and resorbable polylactic acid, it is widely recognized that polyester mesh elicits a robust foreign body response and triggers long-lasting chronic inflammatory reactions. [50, 51] Furthermore, polylactic acid is also known to induce a persistent inflammatory response since, being degraded in 3–4.5 years after implantation, further lymphohistiocytic involvement with persistent inflammatory reaction until complete reabsorption occurs [52]. This enduring, nearly permanent reactive nature of Progrip has also been histologically confirmed in implant specimens removed several months after surgery (Fig. 5, 6). In contrast, despite also being deployed fixation-free, ProFlor operates on an entirely different principle. Firstly, the 3D scaffold wholly and permanently fills the hernia defect, thereby preventing the possibility of subsequent engagement of the hernia opening by protruding visceral tissue. Furthermore, the dynamic response of ProFlor’s 3D core encompasses some proprietary features that significantly characterize this device as a different player in the domain of inguinal hernia repair. Central to this innovation is the scaffold’s dynamic adaptation to the movements of the inguinal region, facilitated by its inherent capacity for compression and relaxation in harmony with inguinal motion. This physiological compliance distinguishes ProFlor from traditional hernia meshes. The dynamic responsiveness of this 3D scaffold may contribute to the activation of tissue growth factors, potentially supporting the formation of new muscle bundles, blood vessels, and nerves within the scaffold structure (Figs. 7, 8, 9), as observed in previous scientific investigations. [29–34]

### Clinical outcomes and biological mechanisms

The comparative assessment of the outcomes of the two techniques revealed differences in terms of operative time length and intraoperative complications. The disparity in early postoperative pain was marginal, although ProFlor was better tolerated by patients. However, the substantial differences emerged from the evaluation of other data closely linked to the inherent physical composition of the tools used in the study. Actually, analyzing the chemical, structural, and physical characteristics of the two devices aids in objectively understanding the outcomes detailed in the results of the current investigation. The notable discrepancies in results between the two devices underscore a statistically significant impact on outcomes, such as seroma formation, discomfort, and chronic pain. This difference can be attributed to the distinct biological and mechanical behavior exhibited by each device. Indeed, the widely recognized long-lasting inflammatory response associated with Progrip serves as the underlying cause of adverse events observed in the early postoperative stage, such as seromas. Furthermore, discomfort and chronic pain experienced in the mid and long term can be attributed to excessive inflammation, leading to an augmented fibrotic reaction that stiffens the mesh. The increased ingrowth of rigid scar tissue can potentially entrap the inguinal nerves located in the posterior aspect of the inguinal backwall, which may contribute to the development of chronic pain syndrome. In the ProFlor group, minimal instances of seroma and mild short-term discomfort were observed, with no reports of chronic pain. Evidence indicates that the inflammatory response to ProFlor is mild and limited to the early stages following implantation, suggesting a favorable biocompatibility profile [25, 26]. This may help explain the low incidence of seromas, which were observed exclusively in the short term. As for the discomfort reported in the mid and long term by the patients and assessed with the CCS scoring system, it commonly arises from the incorporation of stiff fibrotic scar tissue that tends to harden over time and can cause the often from patients reported unpleasant rubbing on the surrounding groin tissue during movement. However, owing to the dynamic nature of ProFlor, which avoids the formation of stiff scar tissue and instead promotes a probiotic response, discomfort was absent in this subset of patients. Remarkably, all patients were able to resume their daily activities practically pain-free within a few days postoperatively. The absence of chronic pain reported in the ProFlor group can be attributed to a similar explanation. Despite both Progrip and ProFlor being placed fixation free, thereby eliminating the risk of nerve entrapment following fixation, only Progrip patients experienced chronic pain. It seemingly resulted from uncontrolled fibrotic apposition around the mesh and neighboring tissue close to inguinal nerves, which may result prone to the exuberant proliferation of hard tissue that could lead to neural compression. In contrast, with ProFlor, the ingrowth of viable, fleshy tissue avoids the risks of nerve entrapment. This fundamental difference between the two devices helps elucidate the absence of this dreaded complication in the ProFlor group.

### Impact of defect closure on recurrence

Concerning the management of hernia defects in the herniated groin, some researchers propose suturing the hernia opening as a method to reduce the risk of recurrence. [21, 53] However, closing the hernia defect with sutures applied to the inguinal musculature is unphysiological and should generally be avoided. The inguinal region’s dynamic nature means that tight knotting of sutures disrupts natural movements, causing mechanical stress and significant postoperative pain due to the high sensitivity of the musculature to trauma. This pain is often compounded by reflexive muscle contractions triggered by the sutures, creating a cycle of discomfort and spasm [7–15]. Moreover, the stress imposed by tightly knotted sutures can lead to micro-tears in the muscle fibers during movement, resulting in bleeding, hematomas, and prolonged inflammation [16, 17]. These complications delay recovery and increase the risk of chronic pain or nerve-related syndromes, particularly when muscle fibers are repetitively strained over time [7, 12]. To minimize these risks, defect closure strategies should respect the natural biomechanics of the inguinal region. Getting back to the outcomes of this investigation, another remarkable disparity between the two groups is the rate of recurrences. Actually two patients in the Progrip group reported recurrence, while no recurrences were recorded in the ProFlor group. It is noteworthy that these recurrences in the Progrip group occurred in hernia defects larger than 35 mm, manifested within 12 months postoperatively likely following mesh invagination into the defect over time. It should also be stressed that in the Progrip approach, consistent with the traditional flat mesh repair concept, the inguinal gap remains patent, whereas ProFlor is specifically designed to fully and permanently obliterate the defect. This underscores the importance of effectively managing the hernia defect since the permanent obliteration makes a significant difference in preventing recurrences.

### Future directions in hernia repair

In light of the findings presented, this study highlights key insights that should be considered when contemplating the future of inguinal hernia repair approaches. Progrip, with its innovative fixation-free solution involving adhering surfaces, undeniably represents a significant advancement in the flat mesh repair concept. However, this type of mesh is still subject to the inherent limitations of the traditional method, particularly regarding the quality of biological response and the intrinsic long-lasting inflammatory reaction. This deficiency may have adverse effects on the physiology of the groin and, in cases of exuberant fibrotic proliferation, can lead to unpleasant complications such as discomfort and chronic pain. Lastly, it is worth noting that the treatment model embodied by Progrip, like the conventional concept of reinforcing the abdominal wall through fibrotic apposition on the mesh, results inconsistent with the degenerative genesis of inguinal hernia disease given that there is neither tissue regeneration nor the restoration of the integrity of the inguinal barrier. In contrast, the concept introduced by ProFlor, which capitalizes on the biological impact of dynamic forces exerted on a receptive scaffold, marks a significant advancement in our comprehension of tissue regeneration. This discovery proves that merely altering the design of a device to confer a different physical response can result in a fundamentally distinct biological outcome. In essence, using conventional biocompatible material, such as polypropylene, and modifying by design the physical characteristics of the from static to dynamic responsiveness, transforms the biological response from the foreign body granuloma formation, typical of flat and static meshes, to a genuine regenerative effect mediated by specific growth factors. This revelation highlights the immense potential for innovation in achieving therapeutic outcomes through thoughtful design considerations in medical devices.

## Conclusion and study limitations

In this randomized study, compared to ProGrip, ProFlor was associated with a decrease of postoperative pain as well as lacking chronic pain and recurrences. In light of the reported evidences, the 3D design of ProFlor addresses the limitations of traditional flat mesh technique. It likely embodies a promising paradigm shift in inguinal hernia repair, emphasizing regeneration over reinforcement, eliminating chronic pain while promoting tissue regeneration.

However, the study has several limitations that warrant consideration. First, this was a multicenter study with a relatively small sample size, which may limit the generalizability of the findings. Second, the follow-up period, although sufficient to detect early and mid-term outcomes, may not fully capture long-term complications or recurrences. Third, the open-label design may introduce bias, as neither patients nor investigators were blinded to the interventions. Finally, the study did not include a cost-effectiveness analysis, which could be important for evaluating the practical application of these devices in diverse healthcare settings.

Despite these limitations, the findings highlight key insights that could improve the management of inguinal hernias. Further multicenter studies with longer follow-up durations and larger sample sizes are needed to validate these results and establish the long-term efficacy of Progrip and ProFlor. Encouraging reflection on this evidence within the surgical community may pave the way for enhanced treatment outcomes in hernia repair.

## Supplementary Information

Below is the link to the electronic supplementary material.

Supplementary file1 (MP4 27137 KB)

Supplementary file2 (DOC 218 KB)

Supplementary file3 (DOCX 71 KB)

## Data Availability

The datasets used and/or analyzed during the current study available from the corresponding author on reasonable request. All data generated or analyzed during this study are included in this published article.

## Declarations

### Research involving human and animal right participants

The investigation has been carried out in accordance with the Declaration of Helsinki for experiments involving humans. Informed consent was obtained for experimentation with human subjects.
