# Supplementary material for: Progrip versus ProFlor: two fixation-free devices for laparoscopic inguinal hernia repair—the Pro/Pro study, a randomized clinical trial
Source: Surg Endosc. 2025 Apr 1;39(5):3113–26. doi: 10.1007/s00464-025-11680-x (PMC12041049; doi:10.1007/s00464-025-11680-x)
Supplement: Supplementary file 3 — Supplementary file3 (DOCX 71 KB) [file 464_2025_11680_MOESM3_ESM.docx]

| **Protocol title** | Laparoscopic repair of inguinal hernia with flat mesh versus three dimensional scaffold.  Progrip versus ProFlor: two fixation free devices for laparoscopic inguinal hernia repair. The Pro/Pro Study, a randomized clinical trial. |
| --- | --- |
| **Acronym** | Pro/Pro Study, a randomized clinical trial. |
| **Developer** | Unit of Laparoscopic Surgery – University Hospital Policlinico of Palermo |
| **Coordinating center** | Unit of Laparoscopic Surgery – University Hospital Policlinico of Palermo |
| **Lead Researcher** | Prof. Antonino Agrusa; Dott. Giuseppe Di Buono |
| **Units involved** | Unit of Laparoscopic Surgery – University Hospital Policlinico of Palermo - Italy  Unit of General Surgery – University Hospital Policlinico of Catania - Italy  Unit of General Surgery – University Hospital of Cagliari - Italy |

| **Type of study** | **X** randomized clinical trial   - Observational retrospective study - Observationa retrospective and prospective study - Case-control study - Descriptive study (case-report, case series) - cross-sectional study - other: …………………………   object of the study:   - medical devices/ medical software - Questionnaire (e.g. quality of life)   󠇯X surgical procedures   - Other:…… |
| --- | --- |
| **Data collected in the study** | **󠄒** images and videos  󠄇 prospective clinical data from patients/clinicians  questionnaire to the patients  clinical data of the follow up  histopathological examination |

1. **Rationale and aim of the study**

A wide range of techniques and implants are used for inguinal hernia repair, with flat mesh reinforcement proving effective in reducing recurrence rates seen in pre-prosthetic methods. Over the past two decades, laparoscopic approaches—TAPP and TEP—have gained traction, particularly for recurrent or bilateral cases. However, these techniques present challenges related to anatomical access, prosthetic selection, and fixation methods.

For large hernia defects, laparoscopic repairs often require larger, heavier meshes with multiple fixation points to prevent invagination under intra-abdominal pressure. This increases the extent of peritoneal dissection and the risk of complications, including fibrotic adhesions, postoperative pain, and chronic discomfort. Common fixation methods, such as metallic or resorbable tacks, may disrupt groin biomechanics, contributing to pain and potential implant displacement in defects exceeding 2 cm. Such issues are a leading cause of recurrence in laparoscopic inguinal hernia repair.

To overcome these challenges, research has focused on developing fixation-free implants. One such advancement is Progrip, a self-fixating bicomponent mesh made of monofilament polyester with a resorbable polylactic acid gripping system. It allows for secure placement without fixation devices, reducing nerve-related complications and chronic pain risk. However, Progrip relies on scar tissue formation for groin reinforcement while leaving the hernia defect itself unclosed.

In contrast, ProFlor introduces a dynamic, 3D regenerative scaffold designed to permanently close the hernia opening. Made of polypropylene, this spring-like device requires no fixation, adapts to groin movements, and promotes a biologically favorable response.

This randomized clinical trial compares laparoscopic inguinal hernia repair outcomes using ProFlor versus Progrip in adult patients with bilateral primary hernias.

- Patients underwent scheduled clinical evaluations at 7 days, 15 days, 1 month, 6 months, 12 months, and 24 months postoperatively.
- The primary objective was to assess complications such as recurrence, hematoma, seroma, testicular swelling, infection, prosthesis displacement, or other adverse events.
- Pain was evaluated using the Visual Analogue Score (VAS) in the early postoperative period (up to 4 weeks).
- Long-term assessment (beyond 4 weeks) utilized the Carolinas Comfort Scale (CCS) to measure pain, discomfort, and other postoperative symptoms.

# Patients selection

Inclusion criteria:

- Patients competent to give consent
- aged between 18 and 85 years,
- diagnosis of bilateral inguinal hernia

Exclusion criteria:

- Recurrent inguinal hernia
- Incarcerated inguinal hernia
- Hernia not in the inguinal area
- Signs of obvious local or systemic infection
- ASA score > 4
- Presenting with unstable angina or NYHA class of IV
- Pregnant
- Active drug user
- Immunosuppression, chemotherapy
- Chronic renal insufficiency
- Abdominal ascites
- Infection in area of the surgical field
- BMI >34

**Primary endpoint: overall incidence of postoperative adverse events**

**Secondary endpoints: evaluations of postoperative pain, discomfort, and quality of life.**

The sample size was calculated based on the review of existing literature concerning laparoscopic inguinal hernia repair and our preliminary data regarding the rate of overall postoperative complications. This percentage ranges from 1% in the case of recurrences up to 35% in the case of seromas. On the basis of these results, the power analysis revealed that a minimum of 74 cases of hernia repair for arm would be needed to detect significant differences of the clinical outcomes.

**Duration of the study: 36 months**

**Adverse events**

Patient care (diagnostics, operative care and follow-up) is performed as standard hospital practice. The meshes used in the trial are CE marked standard mesh already used in routine practice, which means that adverse events are not to be expected. However, all adverse events observed during the study will be recorded and published as part of the results of this trial. Major adverse events will be reported to the principal investigator.

**Publication of study results**

The results from this trial will be published in a medical journal.

**Insurance coverage of the participants**

Participants will not be exposed to any additional risk beyond normal laparoscopic inguinal hernia surgery. Patients are covered by standard patient malpractice insurance.

**Cost estimate, funding and resources.**

The surgeries will not incur additional costs for the operating units. Postage and copying costs are covered by the clinic´s research funds. No additional staff are needed.

No funding will be accepted from external funders (for example mesh companies)

**Trial register and confidentiality**

Data will be entered into electronic format (Excel table, SPSS table) and protected by password in the hospital’s computer.

The trial is registered in ClinicalTrials.gov. with number NCT06556498.

**References**

1. Bracale U, Melillo P, Pignata G, Di Salvo E, Rovani M, Merola G, Pecchia L. (2012) Which is the best laparoscopic approach for inguinal hernia repair: TEP or TAPP? A systematic review of the literature with a network meta-analysis. Surgical Endoscopy. Dec;26(12):3355-3366
2. Schmedt CG, Sauerland S, Bittner R (2005) Comparison of endoscopic procedure vs Progrip and other open mesh techniques for inguinal hernia repair: a meta-analysis of randomized controlled trials. Surg Endosc 19:188-189
3. Amato G, Agrusa A, Calò PG, Di Buono G, Buscemi S, Cordova A, Zanghì G, Romano G. Fixation free laparoscopic obliteration of inguinal hernia defects with the 3D dynamic responsive scaffold ProFlor. Scientific Reports 2022, 12(1), 18971
4. Heniford TB, Walters AL, BS, Lincourt AE, Walters AL, Colavita PD, Belyansky I, Kercher KW, Sing RF, Augenstein VA. Carolinas Comfort Scale as a Measure of Hernia Repair Quality of Life. Ann Surg. 2018 Jan;267(1):171-176

*Translated and refined from the original file (in Italian) 24.02.2025.*
